# Draft Genome Sequence of *Stenotrophomonas* sp. Strain SbOxS2, an Antimony-Oxidizing Bacterium Isolated from Stibnite Mine Tailing Soil

**DOI:** 10.1128/MRA.01219-20

**Published:** 2020-12-03

**Authors:** Natsuko Hamamura, Nobuyoshi Nakajima, Shigeki Yamamura

**Affiliations:** a Department of Biology, Faculty of Science, Kyushu University, Fukuoka, Japan; b Center for Environmental Biology and Ecosystem Studies, National Institute for Environmental Studies, Tsukuba, Ibaraki, Japan; c Center for Regional Environmental Research, National Institute for Environmental Studies, Tsukuba, Ibaraki, Japan; University of Southern California

## Abstract

The antimony-oxidizing *Stenotrophomonas* sp. strain SbOxS2 was isolated from stibnite mine tailing soil. The draft genome sequence of strain SbOxS2 comprises 4.76 Mbp with 4,211 predicted protein-coding sequences. This genome will provide useful information for characterizing the molecular mechanisms associated with heavy metal resistance within the genus *Stenotrophomonas*.

## ANNOUNCEMENT

The Gram-negative bacterium *Stenotrophomonas* sp. strain SbOxS2 was previously isolated from stibnite mine tailing soil that was highly contaminated with heavy metals (1). Many species in the genus *Stenotrophomonas* are known to tolerate numerous heavy metals (2), including uranium and selenium (3, 4). Antimony (Sb) is a naturally occurring toxic metalloid and has been recognized as an element of emerging environmental concern (5, 6). Recent studies have shown that microorganisms capable of catalyzing redox transformations of Sb are rather widespread in contaminated environments (7–10). Strain SbOxS2, previously designated strain S2 (1), was isolated for its ability to oxidize antimonite [Sb(III)] to antimonate [Sb(V)], which is a less toxic form to biota. Here, we present the draft genome sequence of antimony-oxidizing *Stenotrophomonas* sp. strain SbOxS2.

Strain SbOxS2 was obtained from a mine tailing soil (Ehime, Japan [33°53′N, 133°12′E]) using the extinction dilution technique (1) and grown in minimal Xm medium (11) containing 0.002% (wt/vol) yeast extract with 100 μM Sb(III) (as potassium antimonyl tartrate). DNA was extracted using a MoBio PowerSoil DNA isolation kit (Qiagen). A paired-end library (insert size, ∼350 bp) was prepared using a NEBNext Ultra DNA library prep kit (New England BioLabs). Genome sequencing was performed on the HiSeq X sequencing platform (Illumina, CA, USA) at the National Institute for Environmental Studies and yielded a total of 12,025,268 raw paired-end reads (2 × 150 bp). After removing the low-quality sequences (Q ≤ 13), the sequences were *de novo* assembled in slow mode in CLC Genomics Workbench version 20.0.2 (Qiagen) with default parameters, except for the minimum contig length (500 bp) and word size (30). The resulting 26 contigs had an *N*_50_ value of 666,627 bp and a maximum contig length of 927,761 bp. The draft genome sequence of strain SbOxS2 was 4,760,758 bp long, with 387.0× genome coverage and a G+C content of 65.9%. Annotation was conducted using the Prokaryotic Genome Annotation Pipeline (12) and the Rapid Annotation using Subsystem Technology server version 2.0 (13), resulting in 4,211 predicted protein-coding sequences, 61 tRNAs, and 6 complete rRNAs (1 copy each of 16S and 23S and 4 copies of 5S). BLASTn analysis of the 16S rRNA gene showed that this strain is closely related to other *Stenotrophomonas* strains (sequence identity of >99%), such as Stenotrophomonas maltophilia strain IAM 12423 (GenBank accession number NR_041577.1).

Of the coding DNA sequences (CDSs), a total of 1,560 were classified into 312 subsystem categories, and the most abundant categories included amino acids and their derivatives (*n* = 263 CDSs), protein metabolism (*n* = 191), carbohydrates (*n* = 166), and membrane transport (*n* = 157). Functional annotation of the CDSs indicated the presence of the *ars* operon, associated with As and Sb resistance, and other genes related to resistance to heavy metals (e.g., copper, cobalt, zinc, and cadmium). Like other species in the genus *Stenotrophomonas*, strain SbOxS2 has potential for high levels of tolerance to heavy metals.

The draft genome sequence of *Stenotrophomonas* sp. strain SbOxS2 provides useful information for furthering our understanding of microbial antimony interactions, as well as the molecular mechanisms associated with heavy metal resistance within the genus *Stenotrophomonas*.

### Data availability.

This draft genome sequence was deposited in GenBank under accession number JAAVXD000000000, BioProject accession number PRJNA622630, BioSample accession number SAMN14523790, and SRA accession number SRX8044413.
